# SINE jumping contributes to large-scale polymorphisms in the pig genomes

**DOI:** 10.1186/s13100-021-00246-y

**Published:** 2021-06-28

**Authors:** Cai Chen, Enrico D’Alessandro, Eduard Murani, Yao Zheng, Domenico Giosa, Naisu Yang, Xiaoyan Wang, Bo Gao, Kui Li, Klaus Wimmers, Chengyi Song

**Affiliations:** 1 College of Animal Science & Technology, Yangzhou University, 225009 Yangzhou, Jiangsu China; 2 Department of Veterinary Science, University of Messina, 98168 Messina, Italy; 3 Leibniz Institute for Farm Animal Biology (FBN), 18196 Dummerstorf, Germany; 4 Department of Clinical and Experimental Medicine, University Hospital of Messina, 98125 Messina, Italy; 5 Institute of Animal Science, Chinese Academy of Agricultural Sciences, 100193 Beijing, China

**Keywords:** Retrotransposon, Insertion polymorphism, RIP, SINE, Pig, Molecular marker

## Abstract

**Background:**

Molecular markers based on retrotransposon insertion polymorphisms (RIPs) have been developed and are widely used in plants and animals. Short interspersed nuclear elements (SINEs) exert wide impacts on gene activity and even on phenotypes. However, SINE RIP profiles in livestock remain largely unknown, and not be revealed in pigs.

**Results:**

Our data revealed that SINEA1 displayed the most polymorphic insertions (22.5 % intragenic and 26.5 % intergenic), followed by SINEA2 (10.5 % intragenic and 9 % intergenic) and SINEA3 (12.5 % intragenic and 5.0 % intergenic). We developed a genome-wide SINE RIP mining protocol and obtained a large number of SINE RIPs (36,284), with over 80 % accuracy and an even distribution in chromosomes (14.5/Mb), and 74.34 % of SINE RIPs generated by SINEA1 element. Over 65 % of pig SINE RIPs overlap with genes, most of them (> 95 %) are in introns. Overall, about one forth (23.09 %) of the total genes contain SINE RIPs. Significant biases of SINE RIPs in the transcripts of protein coding genes were observed. Nearly half of the RIPs are common in these pig breeds. Sixteen SINE RIPs were applied for population genetic analysis in 23 pig breeds, the phylogeny tree and cluster analysis were generally consistent with the geographical distributions of native pig breeds in China.

**Conclusions:**

Our analysis revealed that SINEA1–3 elements, particularly SINEA1, are high polymorphic across different pig breeds, and generate large-scale structural variations in the pig genomes. And over 35,000 SINE RIP markers were obtained. These data indicate that young SINE elements play important roles in creating new genetic variations and shaping the evolution of pig genome, and also provide strong evidences to support the great potential of SINE RIPs as genetic markers, which can be used for population genetic analysis and quantitative trait locus (QTL) mapping in pig.

**Supplementary Information:**

The online version contains supplementary material available at 10.1186/s13100-021-00246-y.

## Background

Retrotransposons—a heterogeneous group of genetic sequences that have the ability to be transcribed into RNA, reverse-transcribed into DNA, and inserted into a new site in a genome—account for 30–50 % of mammalian genomes and thus represent major genomic parasites of mammals [1]. Accordingly, they play key roles in the structural organization of the genome, in the orchestration of biological processes, and even in the diversity and evolution of species. Retrotransposons are classified into three main groups: long interspersed nuclear elements (LINEs), short interspersed nuclear elements (SINEs), and long terminal repeats (LTRs), including endogenous retroviruses (ERVs) [2]. In general, the retrotransposon landscape of mammal genomes is dominated by LINEs and SINEs, followed by LTR retrotransposons [1].

Although SINEs, which are transcribed by RNA polymerase III, only occupy up to ~ 10 % of mammalian genomes, they display an extremely high occurrence rate and/or high copy number in genomes because they usually appear as short fragments 150–300 bp long. SINEs typically have three parts: a 5′ head, a body, and a 3′ tail; they are non-autonomous retrotransposons that retrotranspose by hijacking the reverse transcriptases (RTs) and endonucleases of their partner LINEs [3]. Because LINEs and LTRs are large fragments (7–9 kb) and are believed to have a greater ability to disrupt genes and genomes than the shorter SINEs (150–300 bp), they are evolutionarily purged from genomes at a greater rate. Thus, SINEs are believed to be more tolerable for hosts, can co-evolve with host genomes, and can exert a wider impact on the shaping of genes and on genome evolution [4].

SINEs have been found to insert frequently in gene regions, suggesting that they might play important roles in regulating gene activity. Approximately 38 % of SINE insertions overlap with transcribed regions in wheat, and 30 % of SINE insertions overlap with genes in Solanaceae [5, 6]; 65.69 % of the transposable element (TE) insertions found in introns were SINEs in bovine genomes [7]. Around 85–90 % of mouse and human protein-coding genes contain TE sequences in their introns [8], while in pigs, nearly 50 % of retrotransposons are inserted into over 80 % of protein-coding and long non-coding RNA (lncRNA) genes, with SINEs representing the highest insertion frequency compared with LINE and LTR retrotransposons [9]. It has been suggested that SINEs can shape gene and genome evolution by offering exons, splicing sites, and start and stop codons, thus creating novel genes [10, 11]. SINE insertions can play roles in gene regulation by diverse mechanisms: by acting on the promoters, enhancers [12], or transcription factor binding sites [13] of corresponding genes. SINE retrotransposons can also contribute to epigenetic regulation; in fact, SINEs possess a high GC content, which makes them hot spots for DNA methylation, a well-known mechanism related to transcriptional repression [14–16]. Furthermore, SINEs can activate miRNAs by acting as promoters for miRNA synthesis, or as miRNA-binding sites in target mRNAs [17–19], or by regulating gene expression from SINE transcripts [20, 21]. When SINEs accumulate in 3′ UTRs, they influence mRNA degradation by Staufen-mediated mRNA decay [22]. When a SINE is inserted into a lncRNA, it can promote translation of partially overlapping sense protein-coding mRNAs (designated a SINEUP), leading to regulation of the expression of the target gene [23, 24].

Recently, retrotransposon-based markers have been reported extensively and widely used in studies of genetic diversity, phylogeny, genetic mapping, and cultivar identification in plants [25–28]. It is commonly accepted that retrotransposon insertion polymorphism (RIP) markers have high prevalence in genomes and are more informative and polymorphic compared with other marker systems [25, 26, 29, 30]. RIP markers have also been developed for several domesticated animals, including sheep, deer, and chicken [27, 31–33]. Our previous study on pig mobilome annotation revealed that most (80 %) of the protein-coding and lncRNA genes contain retrotransposon insertions in pig genomes, and retrotransposons tend to be enriched in lncRNAs, with nearly half of protein-coding genes generating chimeric transcripts with retrotransposons [9]. Furthermore, this indicated that SINEs are the most widespread retrotransposons in the pig genome, accounting for about 11 % with over 1 million copies [9]. SINEA1–3 represents the youngest subfamily of pig-specific SINEs and reflects the most recent expansion activity during the last 10 million years [9]. These data indicate that SINE RIP markers may be important tools for studying biodiversity and genetics, and even for molecular breeding in domestic animals. In particular, several SINE insertions causing phenotype changes have been reported in pigs, horses, and dogs [34–39]. However, genome-wide SINE RIPs are rarely reported in the study of genetics and breeding of livestock, including pig.

Here, we developed a genome-wide SINE RIP mining protocol and performed genome-level screening by using the assembled pig genomes deposited in the NCBI database (see below); the resulting RIPs were further verified by polymerase chain reaction (PCR) amplification. We also evaluated the genomic coverage and breed distribution of these SINE RIPs, their insertion bias, and applied them for population genetic analysis and for evaluating domestication processes in Chinese pig breeds. We obtained a novel set of highly informative RIP markers, with a wide distribution (average 14.5 SINE RIPs per 1 Mb) and high coverage (36,284) in the pig genome, which display great potential as genetic markers for application in phylogeny and genetic diversity studies as well as in quantitative trait locus (QTL) mapping to benefit the conservation and utilization of local pig genetic resources and modern molecular breeding.

## Results

### Young SINE retrotransposon insertions are highly polymorphic in the pig genomes

**Fig. 1:**
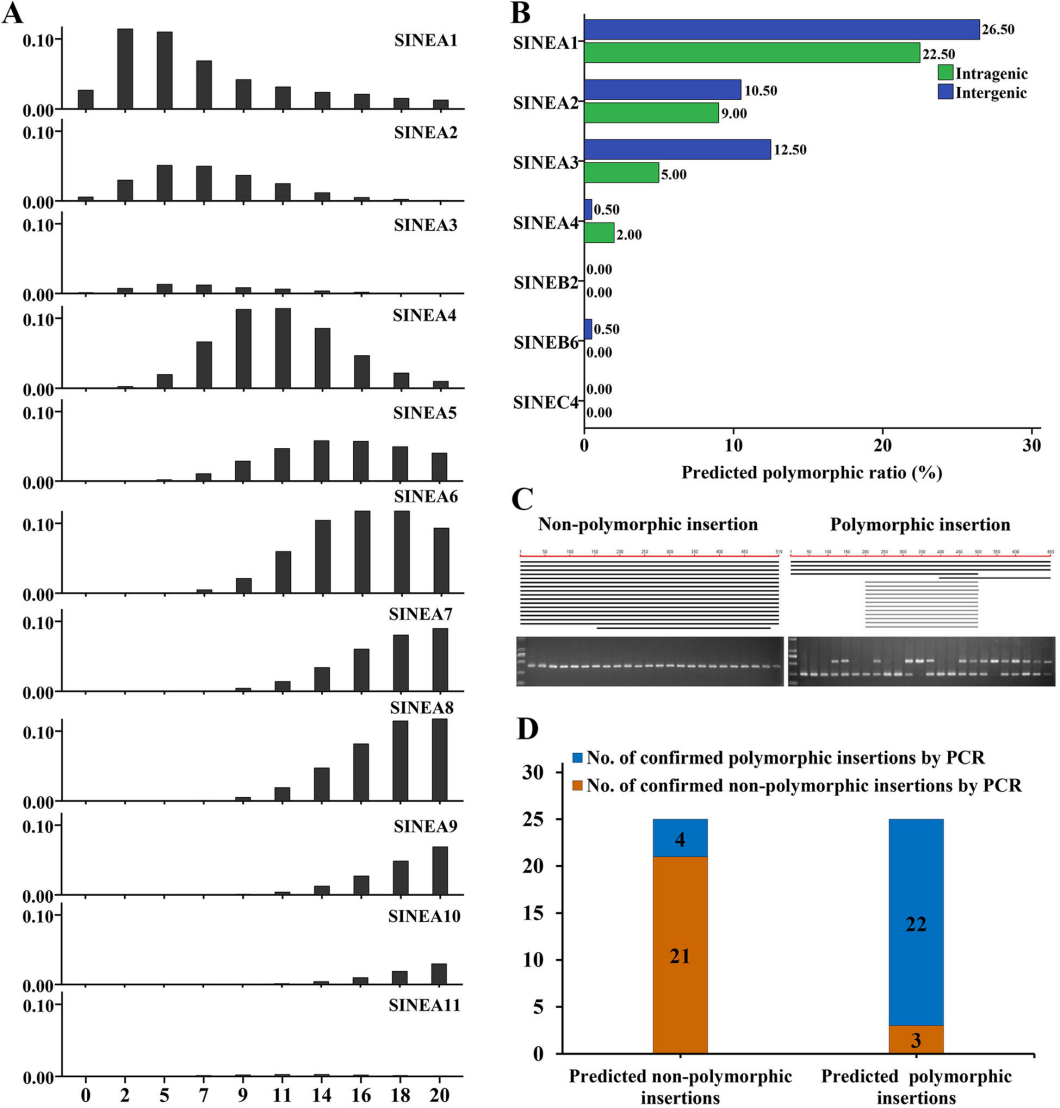
High polymorphic rate of young SINE subfamilies. **A** Insertion ages of SINEA subfamilies. The X-axis represents insertion ages (million years ago, Mya), and the Y-axis represents the genome coverage (%) of SINE subfamilies. **B** Predicted polymorphic ratio of SINE insertions from seven SINE subfamilies representing different insertion ages. **C** Representative results of the BLAST prediction and PCR verification. **D** The results of PCR verification for 25 predicted polymorphic and 25 non-polymorphic insertions from different SINE subfamilies (primers listed in Additional file 2)

Three pig-specific SINE families (SINEA, SINEB, and SINEC), with different evolutionary histories, were identified in a previous study showing that SINEA represents the youngest family with some of its subfamilies still displaying activity in the last 10 million years [9]. Eleven subfamilies of SINEA (A1–A11) were identified previously, and they display high sequence similarity, but with minor differences: SINEA1–SINEA3 have six specific nucleotides, SINEA1 and SINEA2 have two specific nucleotides, while SINEA1 contains the longest polyA sequence (Additional file 1: Fig. S1), which is unique and different from other subfamilies and might act in their transposition activities. Insertion age analysis revealed that SINEA1–SINEA3 displayed activity 2 million years ago (Mya); the activity of SINEA4 was hard to detect in the last 2 million years, while the activity of other subfamilies (SINEA5–SINEA11), SINEB, and SINEC was totally extinct in this period (Fig. 1 A and Additional file 1: Fig. S2). Overall, SINEA1 showed dominant current activity (< 2 Mya), followed by SINEA2, while SINEA3 exhibited very weak current activity, indicating that these subfamilies, particularly SINEA1, might still jump and contribute to genomic variations in pigs.

To investigate the jumping activity of these SINE elements, 1,400 SINE insertions distributed in the intragenic regions, and 1,400 in the intergenic regions in the reference genome from seven SINE subfamilies (SINEA1–SINEA4, SINEB2, SINEB6, and SINEC4), representing different insertion ages, were selected randomly for polymorphism prediction by local BLAST searching as described in the methodology. The predicted polymorphic ratio varied significantly across subfamilies, as expected. SINEA1 showed the highest polymorphic ratios at 22.50 and 26.50 % in intragenic and intergenic regions, respectively. The SINEA2 and SINEA3 subfamilies showed polymorphism rates ranging from 5.00 to 12.50 %, while other subfamilies displayed very low insertion polymorphism rates (< 2 %) (Fig. 1 B and Additional file 1: Table S1). Furthermore, 25 predicted polymorphic and 25 non-polymorphic insertions between a non-reference (Meishan) genome and the reference (Duroc) genome were used to evaluate the accuracy of local BLAST searching by PCR (Fig. 1 C). The accuracies of finding polymorphic and non-polymorphic insertions were 88.00 % (22/25) and 84.00 % (21/25), respectively (Fig. 1 D and Additional file 1: Table S2), indicating that the local BLAST protocol for SINE RIP prediction is highly reliable. These findings confirmed that SINEA1–SINEA3 are still active and can jump within the pig genome, and proved that SINEA1, the youngest element, is very active, and tends to generate highly polymorphic insertions.

### Development of the genome-wide SINE RIP screening protocol

**Fig. 2:**
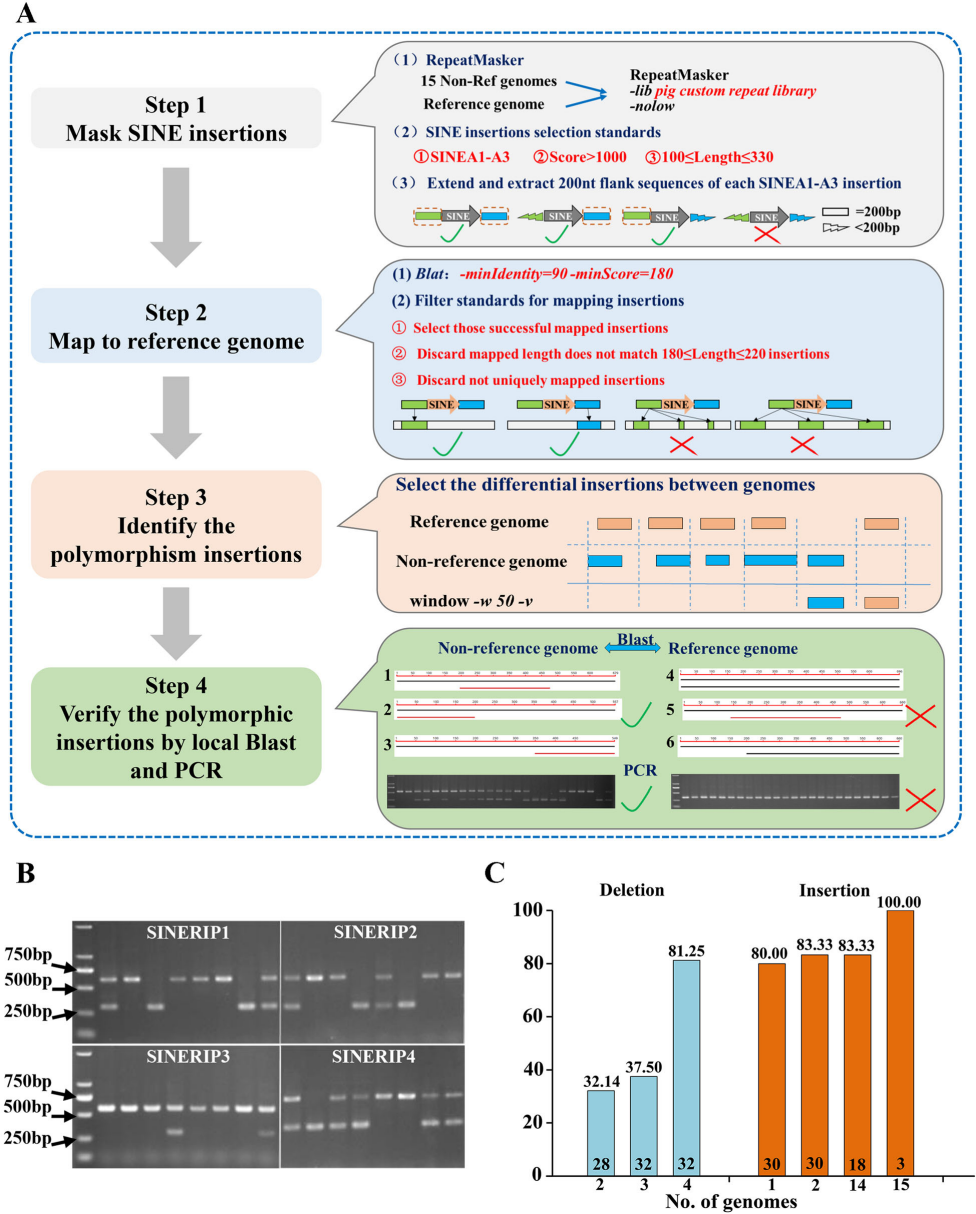
SINE RIP annotation and verification protocol. (**A**) Main steps and methods of SINE RIP annotation. (**B**) Representative electrophoresis results of PCR verification of SINE RIPs. Pooled DNA samples from each breed (the order of the breeds in the gel: Bama, Landrace, Large White, Duroc, Erhualian, Meishan, Sujiang, and Wuzhishan) were used for PCR amplification. (**C**) Results of PCR verification for rare SINE deletion and insertion alleles, (primers listed in Additional file 2). The X-axis represents the number of genomes presenting a certain allele (deletion or insertion relative to the reference genome), The Y-axis represents the percentage positive rate of SINE RIPs confirmed by PCR

To identify SINE RIPs in all assembled pig genomes (15 non-reference and one reference) we developed a genome-wide SINE RIP mining protocol, summarized in Fig. 2 A and described in detail in the methodology. A total of approximately 100,000 SINEA1–SINEA3 insertions in each genome were mapped by RepeatMasker. On average, more than 95 % of these insertions in the non-reference genomes were mapped successfully to the reference genome. Based on the comparison of non-reference and reference genomic SINE insertion positions, we obtained 263,837 putative SINE RIPs from all genomes, which were submitted to local BLAST searching and checked manually for each RIP (Additional file 1: Table S3). The ambiguous SINE RIPs were discarded based on their alignment patterns (Fig. 2 A), and 94,074 SINE RIPs remained for further analysis (Additional file 1: Table S3).

Because the assembly levels of non-reference genomes were lower than the reference genome, the gaps in the non-reference genomes could result in a false positive estimation for the SINE RIP deletion allele. Therefore, we discarded those predicted SINE RIP deletion alleles that were detected only in one non-reference genome, and verified those present in two, three, and four non-reference genomes using PCR (Fig. 2B). As expected, we found a high rate of false positives when the SINE deletion alleles occurred only in two or three non-reference genomes, with accuracies of SINE RIP prediction of only 32.14 and 37.50 %, respectively, so these sites were removed from further analysis. However, the accuracy (81.25 %) was significantly improved when SINE RIP deletions were detected in four non-reference genomes. The SINE RIP insertion alleles identified in one, two, 14, or 15 non-reference genomes were also verified by PCR, and all of them showed high accuracy (> 80 %) (Fig. 2 C; Additional file 1: Table S4). These data indicate that the SINE deletion alleles identified in more than three non-reference genomes and all SINE RIP insertion alleles (one or more non-reference genomes) were at least 80 % accurate.

### Large-scale RIPs generated by SINE jumping in the pig genomes

**Fig. 3:**
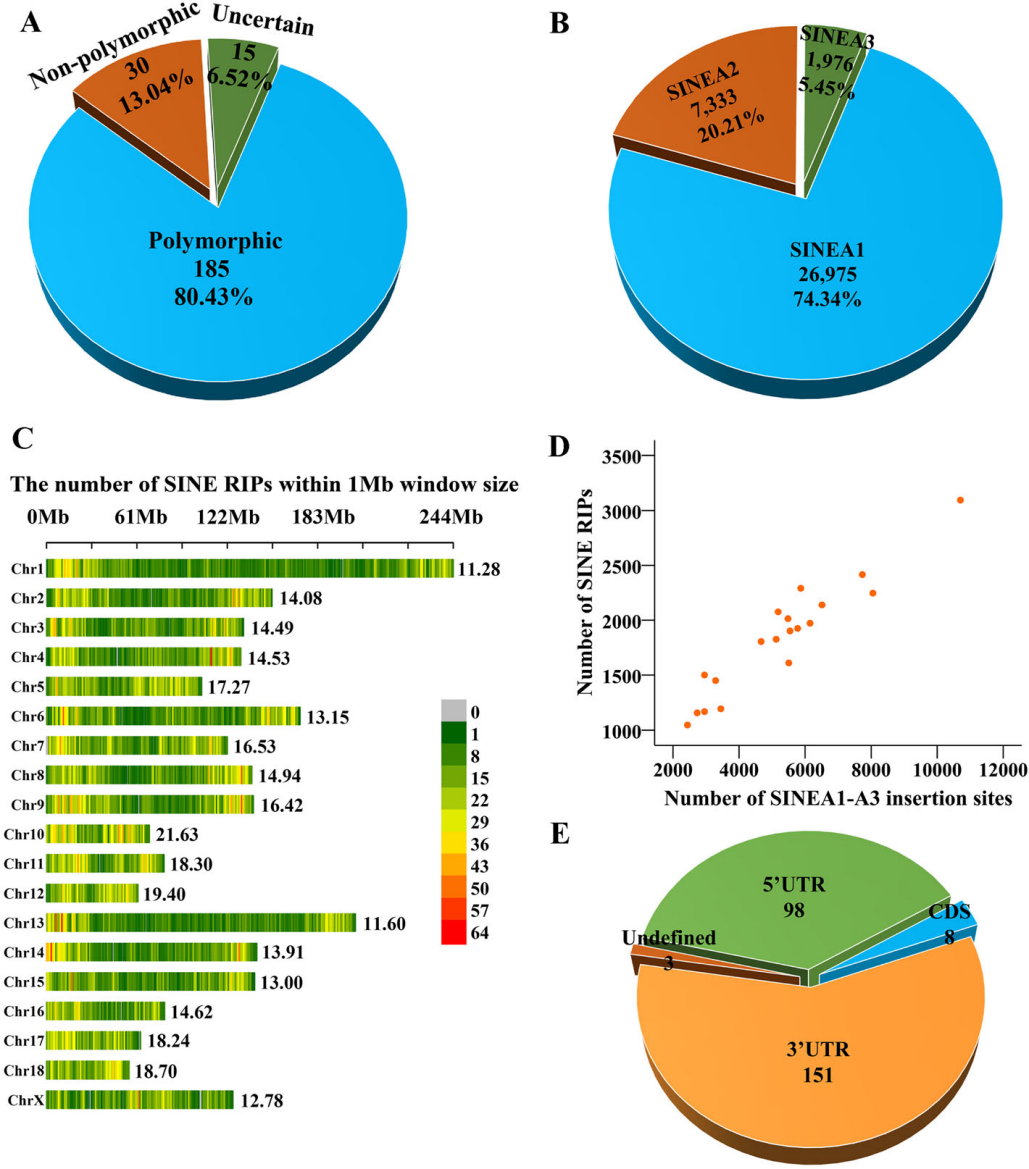
Distribution of SINE RIPs. (**A**) Summary results of PCR evaluation for 230 randomly selected SINE RIPs (primers listed in Additional file 2). (**B**) Distribution of SINE RIPs across SINEA1–SINEA3 subfamilies. (**C**) The distribution and density of SINE RIPs on each chromosome (RIPs/Mb), the number on the right side of each chromosome indicates the density. (**D**) The correlation between the number of SINEA1-A3 insertions and the number of RIPs on each chromosome, Spearman’s correlation value is 0.917 which shows a significantly correlation. (**E**) Distributions of SINE RIPs in the 5′ UTR, 3′ UTR, and CDS regions.

After removing the inaccurate and redundant RIPs, a final total of 36,284 SINE RIPs were obtained at the genome level (Table 1, Additional file 3). Then, 230 SINE RIPs were selected randomly for PCR verification, and 185 RIPs were confirmed as positive, 30 RIPs were false positives, and 15 RIPs were uncertain (Fig. 3 A), resulting in an accuracy of predicting SINE RIPs of > 80 % (Fig. 3 A, Additional file 1: Table S5). Thus, our genome-wide SINE RIP screening protocol was reliable. Overall, 74.34 %, 20.21 %, and 5.45 % SINE RIPs came from the SINEA1, SINEA2, and SINEA3 subfamilies, respectively, which generally corresponds to their age distributions in the genome (Fig. 3B). Furthermore, SINE RIPs were evenly distributed on each chromosome, with an average of 14.5 (range 11.28–21.63) SINE RIPs in each 1 Mb window (Fig. 3 C, Additional file 1: Table S6). While chromosomes 10, 11, 12 tended to be slightly enriched for SINE RIPs (*p* < 0.05/0.01, Fig. 3 C), chromosomes 1, 13 showed a tendency to be slightly depleted of SINE RIPs (*p* < 0.01, Fig. 3 C). The Y chromosome was excluded from analysis because of its multiple repeats, which resulted in difficulties in sequencing and assembly, with too many gaps remaining. Overall, the number of SINE RIPs on each chromosome is significantly correlated with the number of SINEA1-3 insertions (Fig. 3D, Additional file 1: Fig. S3A).

**Table 1:** Summary of SINE RIPs distributed among pig genomes

No. of genomes containing SINE RIPs	No. of insertion alleles	No. of deletion alleles	No. of total SINE RIP alleles
1	11,452	N	11,452
2	4042	N	4042
3	2436	N	2436
4	1575	1730	3305
5	1116	1452	2568
6	783	1478	2261
7	600	1344	1944
8	452	1270	1722
9	265	1380	1645
10	136	1091	1227
11	106	957	1063
12	51	908	959
13	26	620	646
14	18	567	585
15	3	426	429
Total	23,061	13,223	36,284

### Over 65 % of SINE RIPs overlapping with genes

By calculating the genomic positions of each SINE RIP with the biogenic regions, 66.08 % of the SINE RIPs (21,596/32,684) overlapped with the genic regions (NCBI annotated genes and NONCODE annotated lncRNA genes), which represent 23.09 % of the total genes. In all, 51.36 % of the SINE RIPs (16,787/32,684) were found to be overlapping with protein-coding genes, which account for 29.78 % (6,154/20,666) of the total, and most of them (99.09 %) are in introns (16, 635/16, 787). While 13.59 % SINE RIPs (4,443/32,684) overlap with the lncRNA genes, which account for 17.30 % (2,504/14,477) of the total lncRNA genes, most of them (96.89 %) were found to be overlapping with introns (4,305/4,443) as well. 366 SINE RIPs overlap with other non-coding genes (miRNA genes, snoRNA genes and so on), whereas 14,688 SINE RIPs were in the intergenic regions (Table 2). Furthermore, significant biases of SINE RIPs in the transcripts of protein coding gene were observed. A total of 260 SINE RIPs were identified in the exon regions of the protein-coding genes. These SINE RIPs appear to be significantly enriched in the 3′ UTRs (151/260) of mRNAs compared with 5′ UTRs (98/260) and CDS (8/260) (*p* < 0.01, Fig. 3 E; Table 2).

**Table 2:** Intersection of SINE RIPs with genic regions

Type of biogenic regions	No. of SINE RIPs	Percentage^1^ (%)	Denisty(/Mb)	No. of gene contain SINE RIPs
LncRNA gene	4443	100.00	14.75	2504
LncRNA gene-exon	157	3.53	9.43	154
LncRNA gene-first-exon	68	1.53	10.15	67
LncRNA gene-last-exon	89	2.00	9.44	87
LncRNA gene-intron	4305	96.89	14.98	2389
LncRNA gene-intron1	2900	65.27	15.40	1846
LncRNA gene-intron2	1228	27.64	14.10	631
LncRNA gene-intron3	513	11.55	13.61	284
LncRNA gene-intron4	219	4.93	13.17	137
LncRNA gene-intron5	145	3.26	15.88	66
LncRNA gene- 5’ flank (5 kb)	1007	22.66	14.28	946
LncRNA gene-3’ flank (5 kb)	1116	25.12	15.80	1059
Protein coding gene	16,787	100.00	14.67	6154
Protein coding gene-exon	260	1.55	3.11	245
Protein coding gene-CDS	8	0.05	0.22^a^	8
Protein coding gene-5’UTR	98	0.58	4.44^B^	93
Protein coding gene-3’UTR	151	0.90	4.39^B^	147
Protein coding gene-intron	16,635	99.09	15.41	6070
Protein coding gene-intron1	4954	29.51	15.03	2636
Protein coding gene-intron2	3937	23.45	14.78	2102
Protein coding gene-intron3	2726	16.24	14.24	1546
Protein coding gene-intron4	2156	12.84	14.86	1262
Protein coding gene-intron5	1739	10.36	15.04	1094
Protein coding gene-5’ flank (5 kb)	1589	9.47	15.96	1509
Protein coding gene-3’ flank (5 kb)	1435	8.55	14.77	1400
Intergenic	14,688	40.48	14.41	N
Random5kb (N = 20,000)	1380	N	14.07	N
Intragenic	21,596	59.52	14.57	10,347

Note 1: the values used as the denominator to calculate the percentage of SINE RIPs were 4443 for lncRNA genes, 16,787 for protein coding genes, and 36,284 for intergenic and intragenic regions. a, B: indicates *p* < 0.01 with Chi-square test

### Nearly half of all SINE RIPs are common in pig genomes

**Fig. 4:**
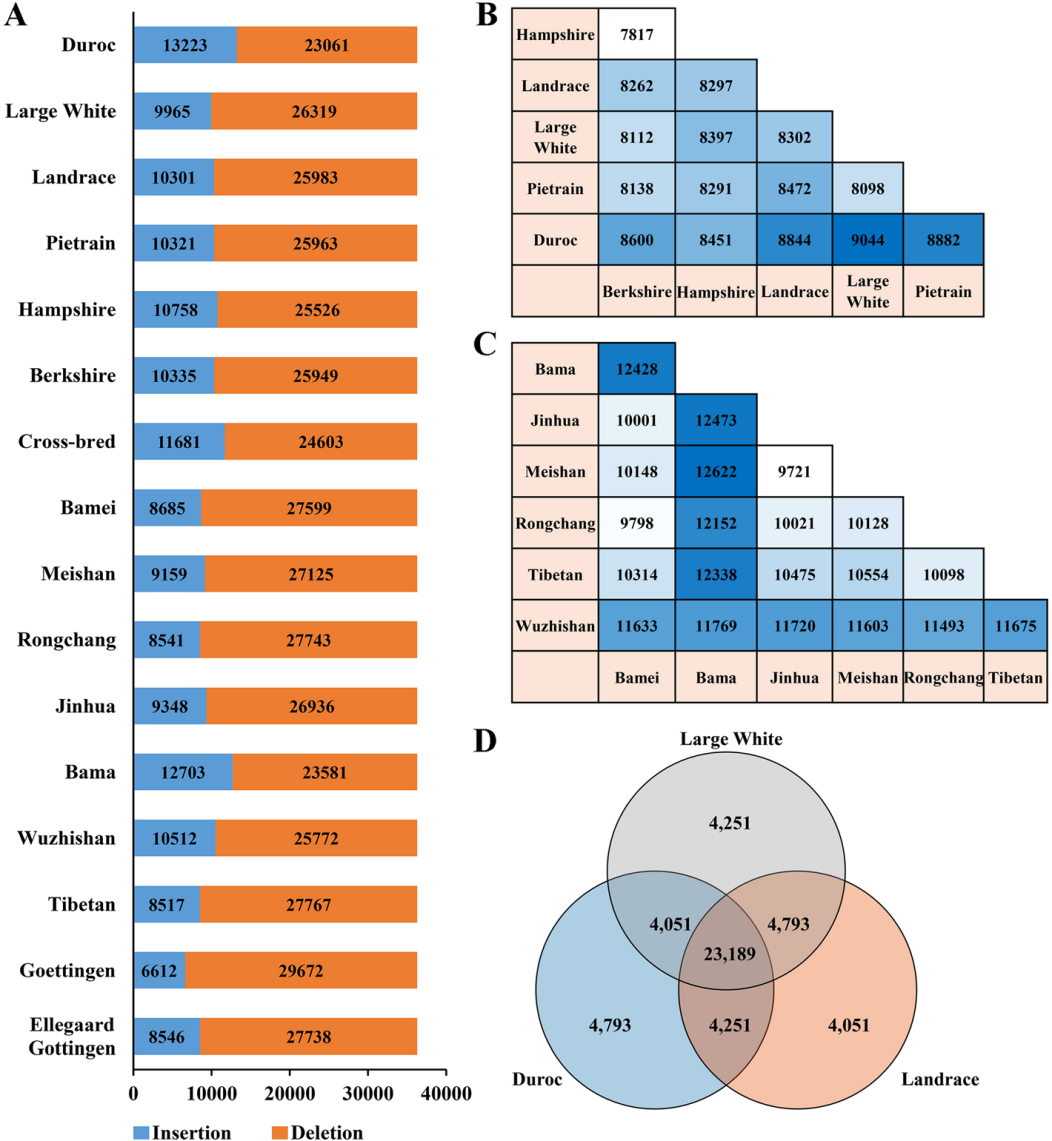
Distribution of the SINE RIP alleles in each genome and between pairs of commercial pig breeds. (**A**) The numbers of deletion and insertion alleles (relative to reference genome) for 36,284 SINE RIPs in each genome. (**B**) Distribution of the differential SINE RIP alleles between different commercial breeds. (**C**) Distribution of the differential SINE RIP alleles between different Chinese native breeds, (**D**) Distribution of the differential SINE RIP alleles between the three most common commercial breeds. The numbers in **B**-**D** refer to the number of the detected differential SINE RIP alleles (presented as deletion or insertion relative to reference genome) between genomes by SINE screen protocol according to the methods.

For the 36,284 SINE RIPs, approximately 10,000 (6,612–12,703) of them appeared as insertion alleles, while the rest of them were identified as deletion alleles in each breed’s genome (Fig. 4 A). Deletion or insertion alleles of the predicted SINE RIPs detected in > 12 or < 4 breed genomes were designated as rare RIPs. In contrast, deletion or insertion SINE alleles present in 4–12 genomes were considered to be common RIPs. Based on this classification, we identified 16,694 common RIPs, representing 46.01 % of all SINE RIPs identified (Table 1), resulting in highly polymorphic sequences in most breeds and with great potential for genetic analysis and QTL mapping. In addition, a pairwise comparison of SINE RIPs across the assembled genomes revealed that, on average, 11,482 differential alleles (range 7,532–14,751) were observed between genomes (Additional file 1: Fig. S3B). Comparison across the commercial pig breed genomes (Duroc, Landrace, Large White, Pietrain, Hampshire, Berkshire) revealed that they exhibited relatively few alleles that differed between genomes, representing about 8,000 SINE RIP alleles, ranging from 7,817 between Berkshire and Hampshire pigs to 9,044 between Duroc and Hampshire (Fig. 4 B). By contrast, the Chinese native pigs displayed more SINE RIP alleles that differed between breeds, with an average of 11,103 (range 9,721–12,622) (Fig. 4 C). Comparison of the most important commercial pig breeds (Duroc, Landrace, and Large White) revealed that 23,189 RIP loci shared the same alleles, with each genome containing about 4,000 (range 4,051–4,793) breed-specific RIP alleles (Fig. 4 D).

### Principal component analysis (PCA) and cluster analysis of the SINE RIPs

**Fig. 5:**
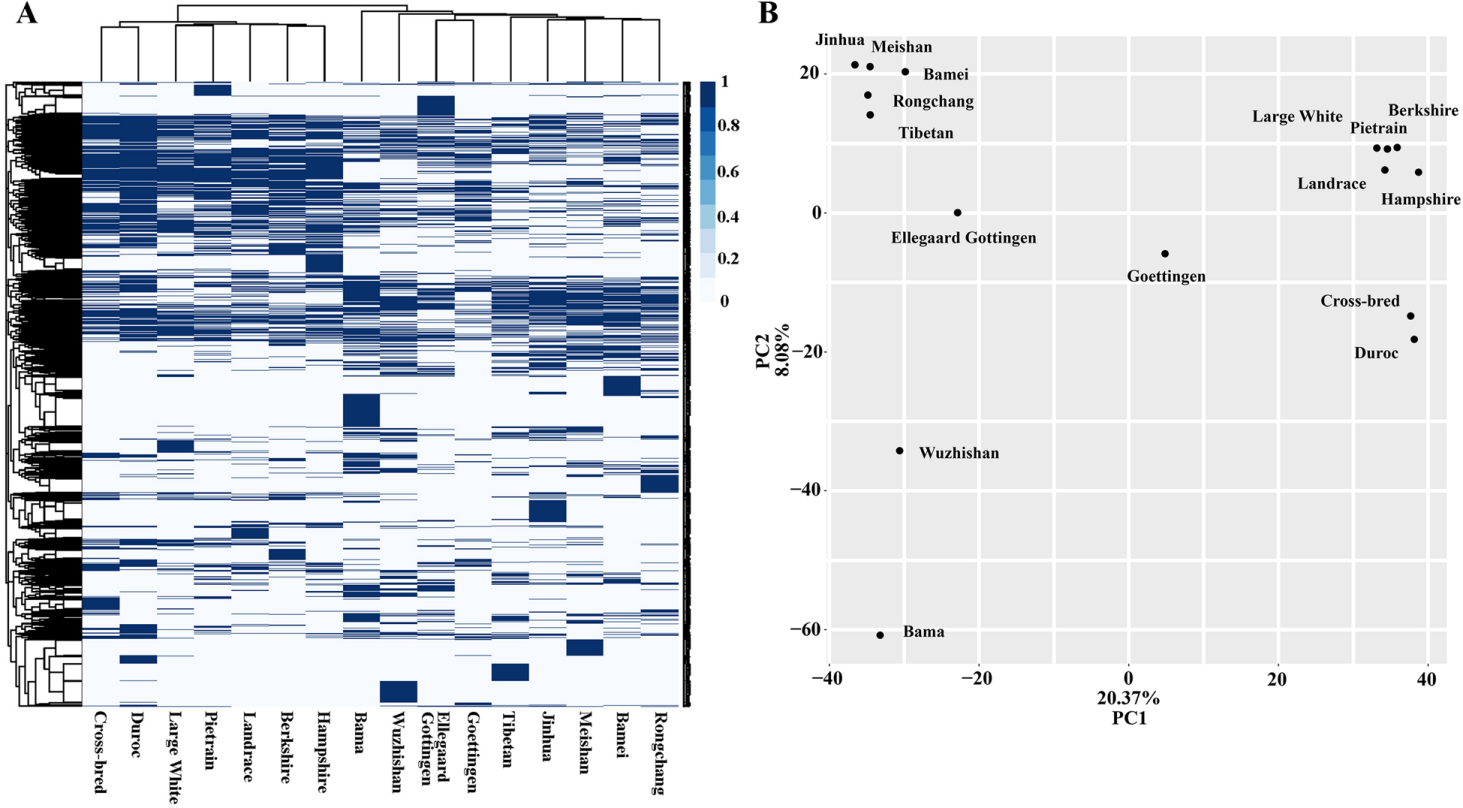
Genetic relationship analysis by heat mapping (**A**) and PCA (**B**) based on all SINE RIPs. The top of (**A**) is the cluster of 16 breeds/lines based on the SINE RIPs and the left of the (**A**) is the cluster of 36,284 SINE RIPs based on their distribution among 16 genomes.

Cluster analysis showed the presence of two main groups of pig breeds, in fact, all Western pigs: Large White, Landrace, Duroc, Pietrain, Hampshire, Berkshire, Duroc, and the cross-breeds form a clade that is well separated from the one comprising all Chinese pigs, including Rongchang, Jianghua, Meishan, Bamei, Tibet, Bama, Wuzhishan, and Göttingen pigs which contained Asian pig genetic material (Fig. 5 A). As expected, the SINE RIP-based clusters were also well supported by PCA (Fig. 5B), in which both clusters are separated horizontally in accord with the direction of maximal variance.

### Analysis of the population structure and genetic diversity of some Chinese native pigs based on SINE RIP molecular markers

To evaluate the potential application of SINE RIPs in population genetic analysis, 16 SINE RIPs were selected to detect polymorphisms in 22 native Chinese pig breeds and in one native Italian pig breed. The PCR analysis revealed that all the markers were polymorphic and biallelic. Detection of SINE RIPs in each breed and their primers are summarized in Additional file 1: Table S7 and Additional file 2.

The Ne statistic per locus ranged between 1.537 (REF-16,266) and 2.000 (ESA1-16), with a mean across loci of 1.765. The expected heterozygosity was higher than the observed heterozygosity at most loci. Observed and expected heterozygosity values ranged from 0.166 (DR-68,328) to 0.468 (REF-3992) and from 0.350 (REF-16,266) to 0.500 (ESA1-16) with overall means of 0.354 ± 0.088 and 0.423 ± 0.055, respectively. While the PIC values, which can reveal the usefulness of a marker in diversity analysis of a breed, are moderately informative for all 16 SINE RIPs (PIC 0.25–0.50), with an overall mean of 0.335 ± 0.031, ranging from 0.288 to 0.375, the negative F_IS_ values (–0.106 ± 0.153), ranging from − 0.315 to 0.328, indicated a low value of inbreeding of each breed detected. The F_ST_ values ranged from 0.117 (REF-14,902) to 0.369 (ESA1-33), with a mean F_ST_ value of 0.252 for all loci, indicating that 74.8 % of the genetic variation was caused by differences between individuals and 25.2 % arose from differentiation between breeds. Agreement with Hardy–Weinberg equilibrium was tested by loci within breeds at *P* < 0.05. For all loci combined, on average about one-third of the breed–loci combinations did not comply with Hardy–Weinberg equilibrium (Additional file 1: Table S7).

**Fig. 6:**
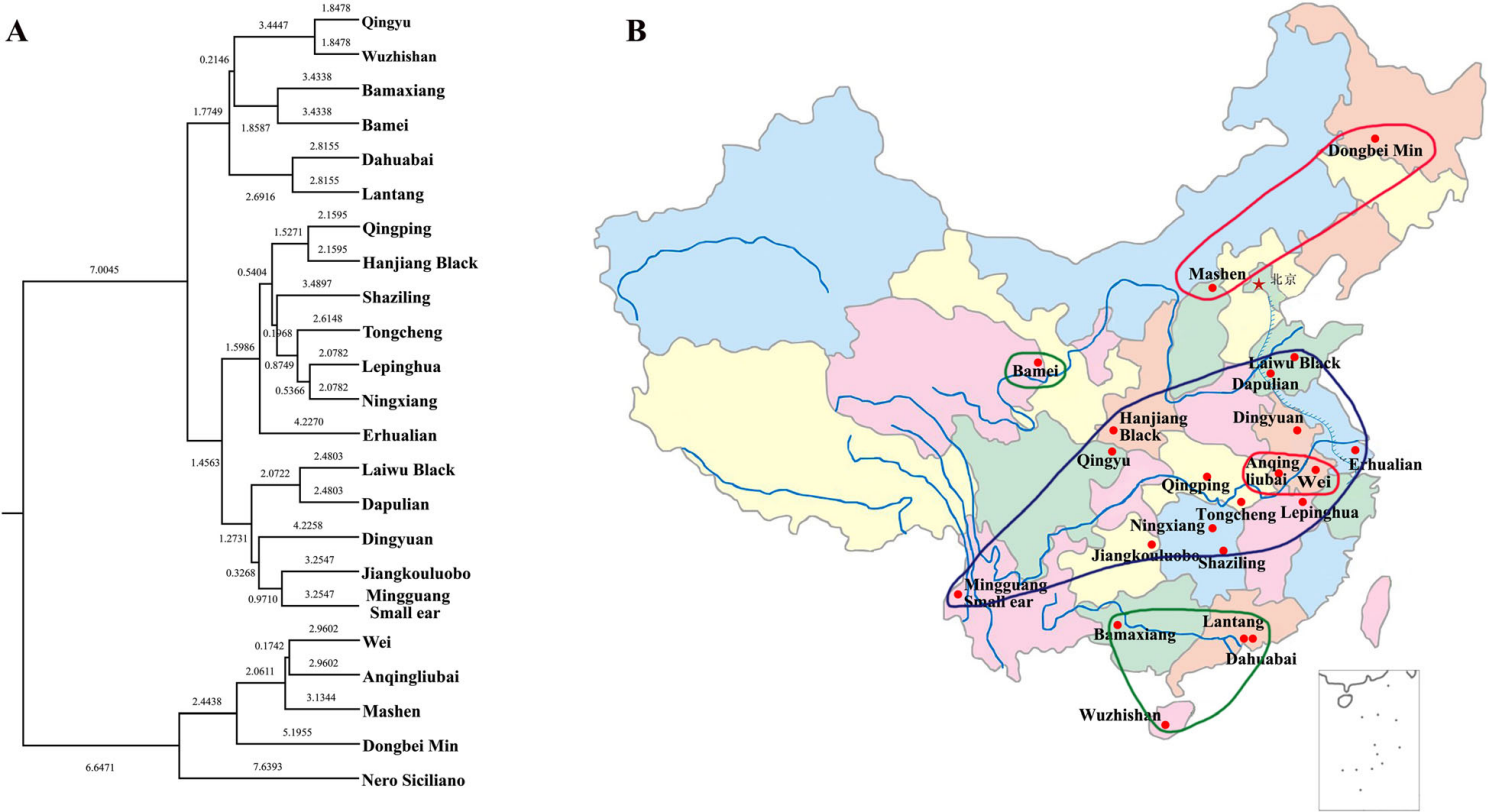
Population genetic analysis. (**A**) Cluster analysis for 23 populations with 16 SINE RIPs. (**B**) Geographic distribution of Chinese native pigs used for analysis, the three coloured lines represent the three clusters based on the phylogenetic tree.PCR primers listed in Additional file 2

The UPGMA method was used to construct a phylogenetic tree (Fig. 6 A) based on Nei’s unbiased genetic distance. This clearly shows three clusters that generally correspond to their geographic locations (Fig. 6B), especially for southern Chinese breeds (Bamaxiang, Wuzhishan, Dahuabai, and Lantang) and most pig breeds of central China (Qingping, Hanjiang Black, Shaziling, Tongcheng, Lepinghua, Ningxiang, Erhualian, Laiwu Black, Dapulian, Dingyuan, and Mingguang Small Ear), with the exception of Bamei, Wei, and Anqinliubai. Bamei is a northern Chinese breed, but clustered with the southern Chinese pigs, while Wei and Anqinliubai were separated from their original geographical location (central China) and clustered with the northern Chinese pigs (Mashen and Dongbei Min) and the Italian pig breed (Nero Siciliano pig), which also has the highest genetic distance from Chinese pig breeds.

## Discussion

### SINE RIPs have great potential as genetic markers

Young retrotransposons, which are very recently evolved elements and still retain jumping activity, have been exploited widely in tagging for gene function annotation [40, 41], and as molecular markers for evolution and population genetic studies [26, 42] in plants and humans [43, 44]. A comprehensive profile of genomic RIPs is critical for the development and application of molecular markers in evolutionary and population genetic studies. However, until now, genome-wide RIP profiles have only been well defined for a few animal species, such as for *Drosophila melanogaster, Caenorhabditis elegans*, and *Homo sapiens* [45–47]. Previous studies suggested that every 20 human genomes contain one new insertion of *Alu* elements, which are a group of SINEs identified in primates [48] and this may be an underestimate, as 60,743 RIPs in rice and 16,192 RIPs in human genomes were identified by analysing re-sequencing data [49–52]. In mice, 695 polymorphic ERVs were identified by comparing the genomic sequences of four common mouse strains [53]. Wei and Kirkness [54] identified at least 10,000 polymorphic SINEC_Cf loci in different dog breeds, and Sara et al. detected 81,747 putative polymorphic SINEs from 62 dogs representing 59 breeds [55]. Our previous study revealed that the pig genome harbours multiple young retrotransposon families/subfamilies, and we have demonstrated that some of them can generate polymorphic insertions [9, 35], suggesting that these retrotransposons, particularly SINE retrotransposons, which represent the most abundant and widest distribution in the pig genome [9, 56], have great potential for the development of genetic markers.

Here, we first evaluated the evolutionary dynamics of different subfamilies of SINEA and compared polymorphism frequencies across these subfamilies. Our data suggest that SINEA1–3 are the youngest subfamilies, possessing functional transposition activity in the last 2 million years. SINEA1 displayed the most polymorphic insertions (22.5 % intragenic and 26.5 % intergenic), followed by SINEA2 (10.5 % intragenic and 9 % intergenic) and SINEA3 (12.5 % intragenic and 5.0 % intergenic), which is consistent with the SINEC_Cf data in dogs, where an average 9 % polymorphism rate [55] and 8 % of polymorphic SINEC_Cfs was reported for the Boxer reference genome [57]. However, the current activities of SINEA4 and other subfamilies are very limited and they have very low polymorphic insertions (< 2 %). These data indicate that SINEA1–3 elements, particularly SINEA1, are major mutators of the pig genome and play important roles in generating new variations in individuals, in population differentiation, and in genomic evolution. The contributions of SINEA1 to the formation of local pig and commercial breeds are also worthy of further evaluation.

We conducted large-scale SINE RIP mining in the pig genome by developing a comprehensive screening protocol using the 16 assembled pig genome sequences deposited in the NCBI database. By comparing the SINEA1–SINEA3 insertion differences across these genomes based on this protocol, we identified 36,284 SINE RIPs. The density of these (14.5 SINE RIPs/Mb) is similar to the single nucleotide polymorphisms (SNPs) represented on the widely used Illumina CAUPorcine 50 K SNP microarray. Furthermore, beside the high density of SINE RIPs, our data also show that these are evenly distributed in the pig genome, strongly supporting them as promising molecular markers for genetic analysis. Here, to improve the accuracy of SINE RIP prediction (> 80 %), we applied a strict standard for multiple key steps of the protocol as described in the methodology and confirmed the reliability of the prediction by PCR evaluation. In addition, a large number of pig breed genomes have been re-sequenced, and the sequences have been deposited in the NCBI database, so SINE RIP mining using the re-sequenced data is expected to increase the number of SINE RIPs significantly and is worth further exploration.

### Most SINE RIPs might be involved in gene regulation

It is commonly accepted that retrotransposons contribute extensively to the diversification of gene function by shaping gene structure or by altering gene activity. Our previous study revealed that about 80 % of protein-coding and lncRNA genes contain retrotransposon insertions in pigs [9], and similar annotations were also observed for the bovine, mouse, and human genomes [58–60]. Over 120 cases of genetic diseases have been reported to be associated with retrotransposon insertions in humans [61]. Furthermore, retrotransposons can regulate gene expression by affecting chromatin structure, gene transcription, pre-mRNA processing, or aspects of mRNA metabolism (for a review see [62]). These data suggest that most retrotransposon insertions can alter the activities of nearby genes. Here, our data demonstrated again that most SINE RIPs (over 65 %) overlap with genic regions, and 29.78 % of protein-coding genes and 17.30 % of lncRNA genes contain SINE RIPs. However, SINE RIPs are significantly depleted in the exons of protein-coding and lncRNA genes: thus, only 260 and 157 were detected, respectively. Additionally, SINE RIPs appear to be significantly enriched in the 3′ UTRs (151/260) compared to CDS in mRNAs, which is generally consistent with the insertion preferences of SINEs in the pig, mouse, and human transcripts [9, 63, 64]. These data indicate again that SINE RIP markers may have larger impacts on gene activities and higher application values in research on population genetics, QTL mapping, and molecular breeding than other types of genetic markers.

### Application of SINE RIPs in population genetic analysis

DNA-based molecular markers such as microsatellites and SNPs are very powerful methods for distinguishing between animal genotypes and have been used extensively in the genetic analysis of pigs [65–71]. SNPs are usually biallelic as co-dominant markers, and less informative compared with that of highly polymorphic microsatellites, but this can be compensated for by employing large numbers of markers (e.g., SNP chips) or WGS [72, 73]. Microsatellite markers are co-dominant, multi-allelic, highly polymorphic, relatively evenly spaced throughout genomes, and require low quality template DNA input (10–100 ng); but they are time-consuming and expensive to develop, and require technical expertise or fluorescently labelled primers for simple sequence repeats (SSR) analysis and high-resolution agarose or polyacrylamide gel separation [74–79]. By contrast, SINE RIPs are biallelic, co-dominant, highly polymorphic, give accurate and reproducible results, and exhibit high coverage and an even distribution among mammal genomes, suggesting great potential as genetic markers. Furthermore, unlike SNPs, whose the ancestral allele is usually uncertain, the ancestral allele of a RIP is the allele without the insertion, because retrotransposons are rarely removed from the genome cleanly, and the insertion events can be used to infer the phylogenetic relationship of species/breeds and deduce the evolution history of these lineages more accurately [80].

We applied 16 SINE RIPs in 23 pig breeds for population genetic analysis. As expected, the PIC and observed and expected heterozygosity values estimated by the SINE RIPs, as important parameters of genetic diversity, were lower than predictions based on microsatellite markers [65–71, 81] but similar to estimates based on SNPs [77–79, 82]. This is probably because microsatellite markers are multi-allelic, while SINE RIPs and SNPs are biallelic. In addition, the individual-level and population-level allele frequency of each type of genetic marker has an important impact on its application. The rare and low-frequency genetic variants are routinely excluded from genome-wide association studies (GWAS) because when an allele is present in a few individuals, the statistical analysis used to draw correlations between traits and alleles is not powerful enough to obtain significant results [83, 84]. Genetic variants presenting only in very few populations/breeds also have significantly limited application value in animal genetics and breeding. Here, by excluding the rare and low-frequency alleles of SINE RIPs (alleles present in > 12 or < 4 assembled genomes), we found that about 50 % SINE RIPs are common, indicating that most of these RIP loci are polymorphic in these breeds, and applicable in population genetic analysis.

Mitochondrial DNA sequences, microsatellite markers, and SNP markers have been used to trace the domestication and origins of European and Asian domestic pigs [85–87]. Over the past decade, regions in China, including the Mekong River basin, the downstream region of the Yangtze River, the upper stream region of the Yangtze River, the Tibetan highlands and the lower region of the Yellow River [88–92] have been suggested as regions from which wild boar might have contributed to the domestic pig gene pool, and which may have represented independent centres for pig domestication. Here, heatmaps of the clusters related to breed comparison were also well supported by PCA with the whole array of SINE RIPs (Fig. 5 A, B), consistent with the results of pig evolutionary research and geographical distribution. Clustering using 16 SINE RIPs in 23 breeds is generally consistent with the geographical distributions of Chinese pig breeds, However, a few breeds do not match completely with their geographical distributions. This discrepancy can be explained by gene flow between these regions or breeds; alternatively, these breeds might not originate locally but were imported historically, which is worth further study.

## Conclusions

Our data suggest that SINEA1–3 are the youngest subfamilies in pig genome. SINEA1 displayed the most polymorphic insertions (22.5 % intragenic and 26.5 % intergenic), followed by SINEA2 (10.5 % intragenic and 9 % intergenic) and SINEA3 (12.5 % intragenic and 5.0 % intergenic), These data indicate that SINEA1–3 elements, particularly SINEA1, are major mutators of the pig genome and play important roles in generating new variations in individuals, in population differentiation, and in genomic evolution. Then we developed a genome-wide SINE RIP mining protocol to mine the young SINE insertion polymorphic sites and obtained a large number of SINE RIPs (36,284), with over 80 % accuracy and an even distribution in chromosomes. Nearly half of the RIPs are common in these pig breeds. Over 65 % of pig SINE RIPs overlap with genes, and about one forth (23.09 %) of the total genes contain SINE RIPs. Sixteen SINE RIPs were successfully applied for population genetic analysis in 23 pig breeds. Our experiments have demonstrated the efficiency of the SINE RIP mining protocol and provide evidence to support their potential as genetic markers in pigs as well as in other livestock.

## Methods

### Assembled genomes and gene annotation files used

Sixteen assembled pig genomes: Duroc, Landrace, Large White, Pietrain, Berkshire, Hampshire, cross-bred (Large White _ Landrace _ Duroc), two lines of Göttingen minipigs (Göttingen minipig, Ellegaard Göttingen minipig), Wuzhishan, Tibetan, Rongchang, Meishan, Bamei, Bama, and Jinhua were used for genome-wide screening of SINE RIPs and were obtained from the NCBI whole-genome sequencing (WGS) database (https://www.ncbi.nlm.nih.gov/assembly/). These assembled genomes had an average sequencing depth of 108.80×. The Duroc is the reference genome (Sscrofa11.1) used for the pig, and the other 15 genomes were re-sequencing genomes obtained by next-generation sequencing technology, which are called non-reference genomes here. Seven of them (Duroc, Landrace, Large White, Pietrain, Berkshire, Hampshire and cross-bred) are commercial pigs, seven of them (Wuzhishan, Tibetan, Rongchang, Meishan, Bamei, Bama, and Jinhua) are Chinese native pig breeds, and five of them (Göttingen, Ellegaard Göttingen minipig, Wuzhishan, Tibetan, and Bama) are miniature pigs. Detailed information about these genomes is shown in Additional file 1: Table S8.

The file on lncRNA gene annotation was downloaded from the NONCODE database (http://www.noncode.org/download.php). The Bed format file of lncRNA genes, which represents 17,811 such genes corresponding to Sscrofa10.2, were converted to Sscrofa11.1 by LiftOver (http://genome.ucsc.edu/cgi-bin/hgLiftOver), and finally, the coordinates of 14,477 lncRNA genes were obtained. The coordinates of protein-coding genes (20,666) and exons, the mRNAs (63,568) of protein-coding genes, and the 5′ UTR, 3′ UTR, and coding sequence (CDS) information of protein-coding genes were retrieved from the annotation of Sscrofa11.1 in the NCBI database (https://ftp.ncbi.nlm.nih.gov/genomes/all/annotation_releases/9823/106/).

### Insertion age estimation and multiple alignments of SINEs

The reference genome (Sscrofa11.1) was masked using RepeatMasker [93] (version 4.0.9, -nolow) with the custom repeat library [9]. Then, the diversity (K) value of each subfamily in SINEA was calculated using the calcDivergenceFromAlign.pl tool in the RepeatMasker program. The ages of SINE subfamilies were then calculated according to the formula T = K / 2r (r = 2.2 × 10^− 9^ substitutions/site/year) [94]. Multiple alignments were constructed from the reference sequences of the SINEA subfamilies using ClustalX2 [95] (version 2.0) with default parameters.

### Genome-wide SINE RIP screening protocol

A protocol for the genome-wide screening for SINE RIPs based on the 16 assembled pig genomes was established in this study, of which the main process is shown in Fig. 2 A and divided into four main steps.

*Step 1. Screening SINE insertions in the genomes.* The custom library (including all SINE subfamilies, DNA, LINE, and LTR repeats) was built in advance [9], and used to mask the 15 non-reference and reference genomes using RepeatMasker (-nolow, -lib custom library). Then, insertions masked by three young SINE subfamilies (SINEA1, SINEA2, and SINEA3) with a length of 100–330 bp and mask score > 1000 were kept for further analysis. The 200 bp upstream or downstream flanking sequences of these insertions were extracted using the bedtools [96] (version 2.27.1) *flank* and bedtools getfasta commands.

*Step 2. Mapping to the reference genome.* The flanking sequences of these SINE insertions in the non-reference genomes were mapped to the reference genome using Blat [97] (-minIdentity = 90, -minScore = 180). The mapping results were filtered by a length of 180–220 bp, and insertions with flanking sequences mapping to more than one genomic position were also removed. For insertions that failed to be mapped against the reference genome by the upstream 200 bp flanking sequence, the 200 bp downstream flanking sequences were mapped in the same way, then the results of these two sets were merged. Thereby, each insertion’s information corresponding to the reference genome was obtained from each non-reference genome.

*Step 3. Identification of SINE RIPs.* The differential insertions, designated as putative SINE insertion polymorphisms between the non-reference and reference genomes, were obtained using a bedtools window (-w 50, -v). The SINE insertions from non-reference genomes that did not fall into the same window (SINE insertion site and 50 bp flanking region) as in the reference genome were considered to be putative SINE RIPs.

*Step 4. Verification of SINE RIPs by local BLAST and PCR.* The putative SINE RIPs were manually verified by local BLAST [98]. The sequences, including the 200 bp flanking sequences and the SINE sequence of each putative SINE RIP, were extracted using bedtools getfasta, and aligned using a local BLAST platform (blastn -task megablast -evalue 1.0e-5 -max_target_seqs 1 -max_hsps 1) between the non-reference and reference genomes. After the alignment, those putative SINE RIPs exhibiting the expected alignment patterns between genomes were kept for further analysis (Fig. 2). The SINE RIPs from all genomes were merged with bedtools merge (-s, -d 10) and redundancies were removed; 403 of the predicted SINE RIPs were selected for accuracy evaluation using PCR amplification.

### PCR verification

Twelve domestic pig breeds (Large White, Landrace, Duroc, Meishan, Erhualian, Sujiang, Fengjing, Diannan small-ear, Wuzhishan, Bama, Tibetan and Nero Siciliano) were used for PCR verification of SINE RIP polymorphisms. The Sicilian black pigs were from Italy and other breeds were from China (Additional file 4). From each pig breed, three individual DNA samples were pooled. DNA was isolated from ear samples using MiniBEST Universal Genomic DNA Extraction kits (TaKaRa, Dalian, China). The primer pairs were designed for the up- and downstream flanking regions of RIPs and spanned the SINE insertions. PCR amplifications were carried out in a total volume of 20 µL, containing 40 ng of genomic DNA, 2 ×Taq Master Mix buffer (Vazyme, Nanjing, China) and 10 pmol of each primer. PCR amplifications were carried out using the following method: an initial denaturation at 94 °C for 3 min; 30 cycles at 94 °C for 30 s; 58 °C for 20 s; 72 °C for 30 s; and a final extension of 10 min at 72 °C. Finally, 7 µL of PCR products and 5 µL of DL2000 molecular weight markers were detected by electrophoresis using 1.0 % agarose gels in 1× TAE buffer with a constant voltage of 130 V for 30 min. Gels were stained with ethidium bromide and visualized with ultraviolet fluorescence.

### Intersection analysis

The distribution of these SINE RIPs in the genome and their relationship with genes and biogenic regions were analysed. Only the overlapping sequences of SINE with gene or biogenic regions above 25 bp were considered for further analysis. Some SINE RIPs interacted with more than one biogenic region or gene, so were counted more than once.

### PCA and cluster analysis of the SINE RIPs

Based on the SINE RIPs identified in this study, the R statistics package (version 3.6.3) was used to generate a presence/absence matrix and performed the PCA analysis. On the same dataset, heatmaps and cluster analysis were computed by the use of the R package pheatmap tool (version 1.0.12) [99], using the “Euclidean” distance method for clustering.

### Genetic diversity and population structure analysis

Sixteen SINE RIPs from 16 chromosomes (Additional file 2) and 585 individuals from 23 breeds (Additional file 3) were selected for genetic diversity and population structure analysis. PCR amplification and detection were performed as described above. The genetic parameters—allele/genotype frequency, effective allele number (Ne), observed heterozygosity (Ho), expected heterozygosity (He), Wright’s F-statistics (F_IT_, F_IS_, F_ST_) and Nei’s genetic distance was analysed with Popgen32 (version 1.32)(https://sites.ualberta.ca/~fyeh/popgene_info.html) and polymorphic information content (PIC) was calculated as $PIC=1-{\sum }_{i=1}^{n}{p}_{i}^{2}-{\sum }_{i=1}^{n-1}\sum _{j=i+1}^{n}{2p}_{i}^{2}{p}_{j}^{2}$ based on the PCR results. Finally, a phylogenetic tree was constructed using the unweighted pair group method with arithmetic mean (UPGMA) method with Popgen32.

### Statistical tests

Chi-square test was used to determine differences for the distributions of SINE RIPs on each chromosome, in different genic regions of gene and transcripts, and Spearman’s correlation analysis was performed to reflect the overall correlation between the number of SINEA1-A3 insertions and the number of SINE RIPs using SPSS (version 16.0; Chicago, IL, USA).

## Supplementary Information

**Additional file 1:** **Figure S1**. Alignment of the sequences of SINEA1-A11 subfamilies. The purple box and four-pointed stars indicate the six specific nucleotides in SINEA1-A3 and red box with five-pointed star indicate the specific nucleotides in SINEA1-A2 from other SINEs in SINEA family. **Figure S2**. Insertion ages of SINEB and SINEC families. **Figure S3**. (A) Distribution of SINE RIPs (outer ring) and SINEA1-A3 insertions (inner ring) on each chromosome. The colors show the number of SINE RIPs or insertions per million base pairs, as indicated by the bars on the right. (B) Distribution of the differential SINE RIP alleles between each pair of genomes. **Table S1**. Predicted polymorphic ratio of SINE insertions from different subfamilies located in intergenic and intragenic regions. **Table S2**. Polymorphic ratio of randomly selected polymorphic and non-polymorphic SINE insertions following PCR verification. **Table S3**. Summary of the number of SINE insertions in the protocol used for annotating SINE RIPs. ** Table S4**. Positive ratios of SINE RIPs obtained by PCR verification for rare SINE RIPs. **Table S5**. Positive ratios of the 36,284 SINE RIPs obtained by PCR verification with limited samples. **Table S6**. Density of SINE RIPs in each chromosome. **Table S7**. Characterization of 16 SINE RIPs analysed in 23 pig populations. **Table S8**. The pig genomes used for the SINE RIP screen protocol.

**Additional file 2:**

**Additional file 3:**

**Additional file 4:**

## Data Availability

All data needed to evaluate the conclusions in this paper are present either in the main text or the supplementary materials.
