# Supplementary material for: SINE jumping contributes to large-scale polymorphisms in the pig genomes
Source: Mob DNA. 2021 Jun 28;12:17. doi: 10.1186/s13100-021-00246-y (PMC8240389; doi:10.1186/s13100-021-00246-y)
Supplement: Supplementary file 1 — Additional file 1: Figure S1. Alignment of the sequences of SINEA1-A11 subfamilies. The purple box and four-pointed stars indicate the six specific nucleotides in SINEA1-A3 and red box with five-pointed star indicate the specific nucleotides in SINEA1-A2 from other SINEs in SINEA family. Figure S2. Insertion ages of SINEB and SINEC families. Figure S3. (A) Distribution of SINE RIPs (outer ring) and SINEA1-A3 insertions (inner ring) on each chromosome. The colors show the number of SINE RIPs or insertions per million base pairs, as indicated by the bars on the right. (B) Distribution of the differential SINE RIP alleles between each pair of genomes. Table S1. Predicted polymorphic ratio of SINE insertions from different subfamilies located in intergenic and intragenic regions. Table S2. Polymorphic ratio of randomly selected polymorphic and non-polymorphic SINE insertions following PCR verification. Table S3. Summary of the number of SINE insertions in the protocol used for annotating SINE RIPs. Table S4. Positive ratios of SINE RIPs obtained by PCR verification for rare SINE RIPs. Table S5. Positive ratios of the 36,284 SINE RIPs obtained by PCR verification with limited samples. Table S6. Density of SINE RIPs in each chromosome. Table S7. Characterization of 16 SINE RIPs analysed in 23 pig populations. Table S8. The pig genomes used for the SINE RIP screen protocol. [file 13100_2021_246_MOESM1_ESM.docx]

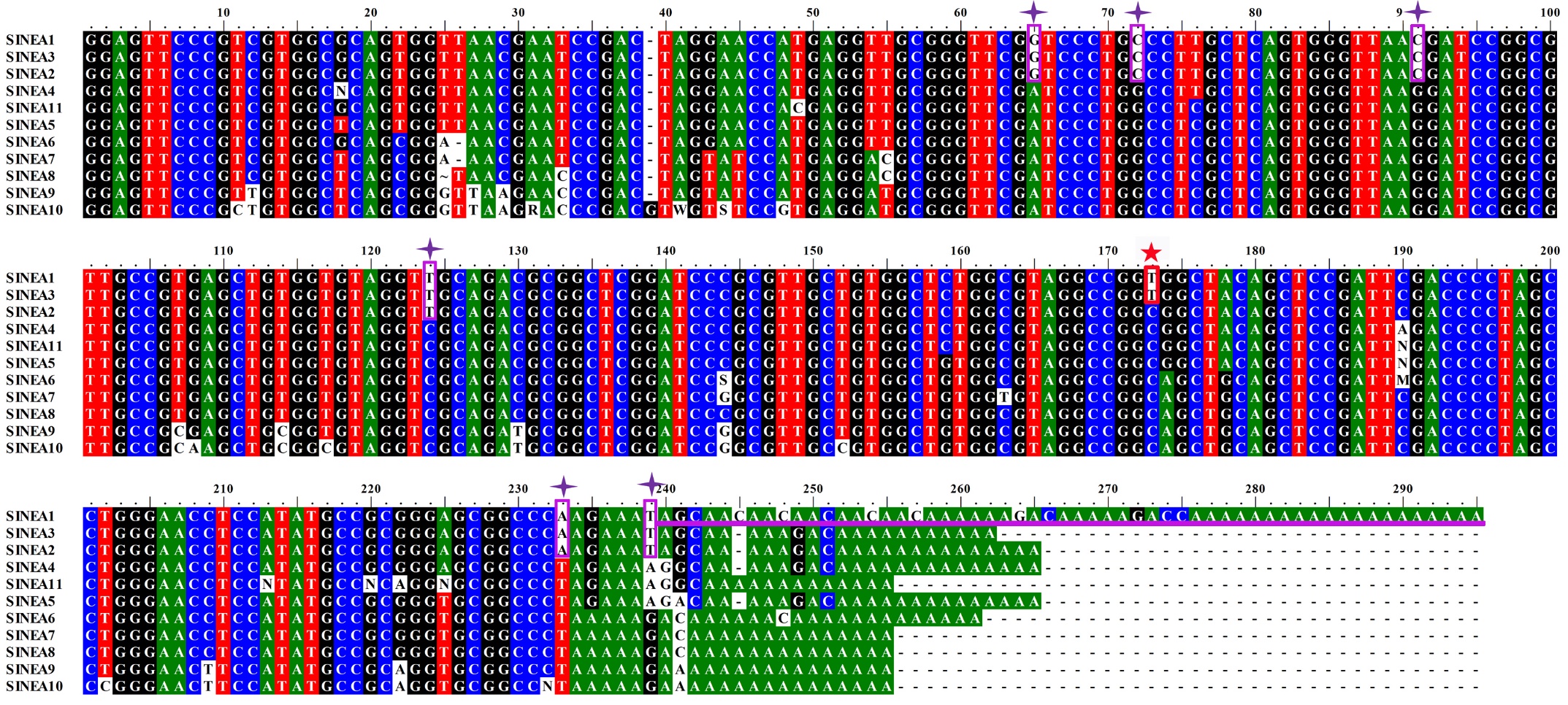


**Fig. S1.** Alignment of the sequences of SINEA1-A11 subfamilies. The purple box and four-pointed stars indicate the six specific nucleotides in SINEA1-A3 and red box with five-pointed star indicate the specific nucleotides in SINEA1-A2 from other SINEs in SINEA family.


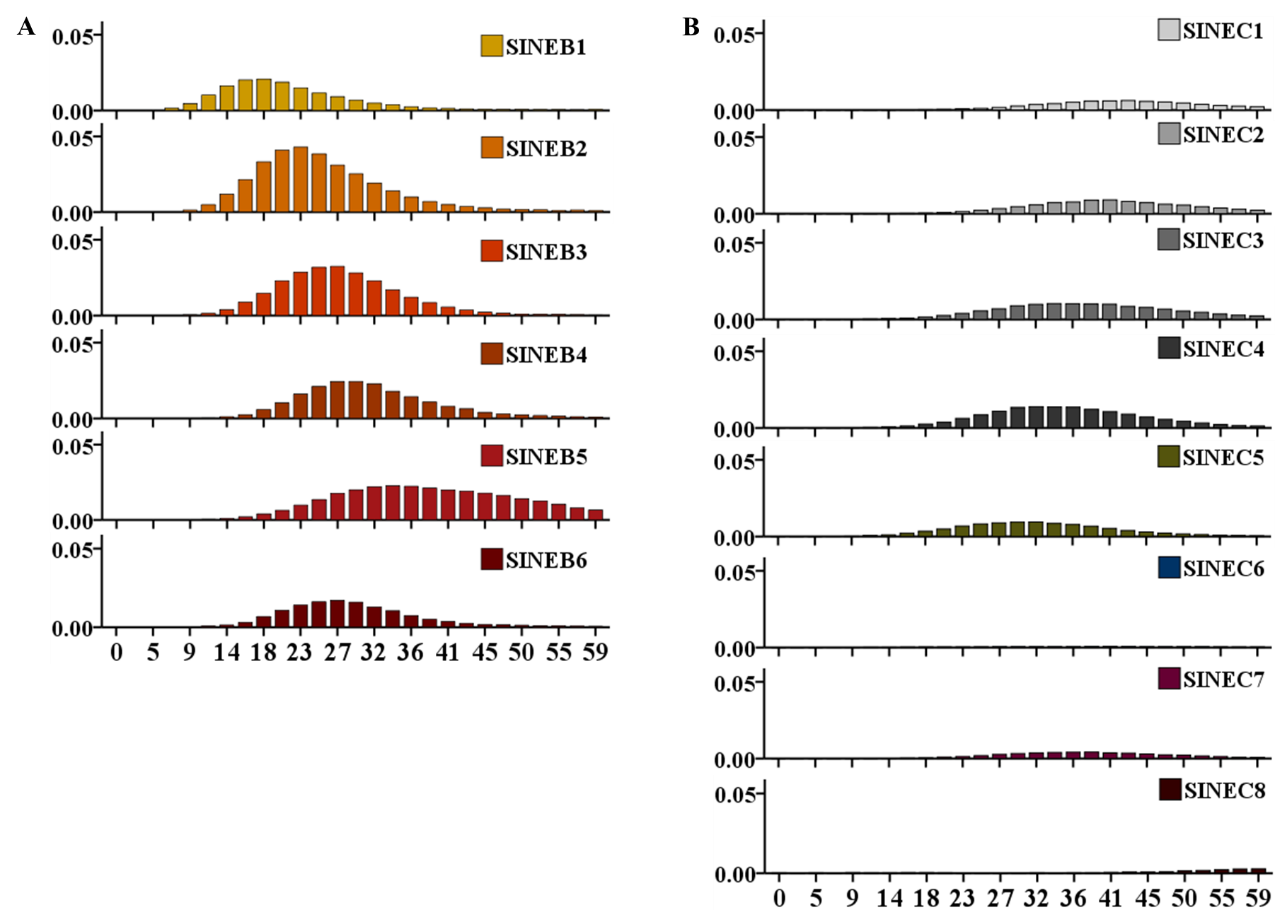


**Fig. S2.** Insertion ages of SINEB and SINEC families.


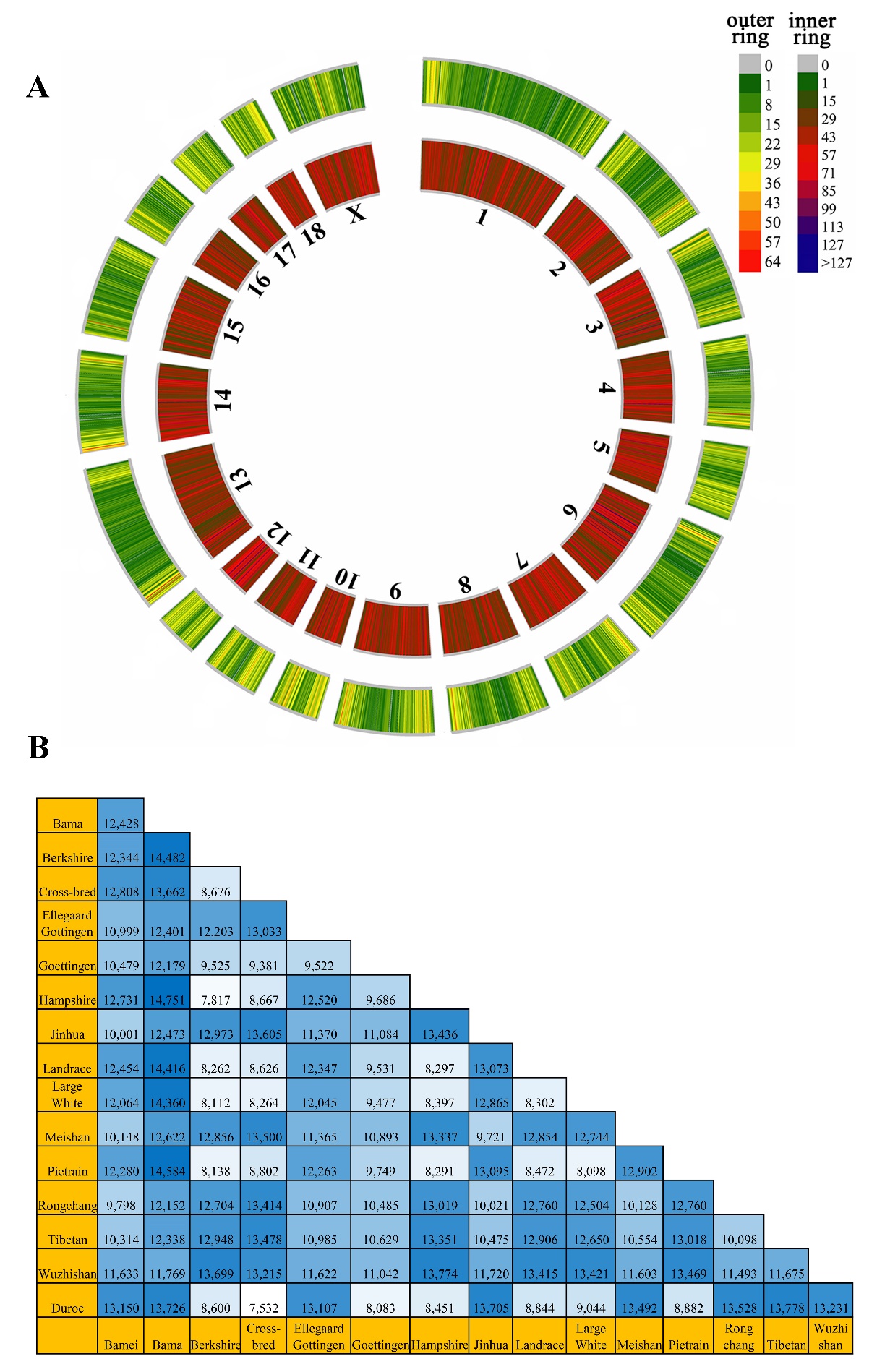


**Fig. S3.** A. Distribution of SINE RIPs (outer ring) and SINEA1-A3 insertions (inner ring) on each chromosome. The colors show the number of SINE RIPs or insertions per million base pairs, as indicated by the bars on the right. B. Distribution of the differential SINE RIP alleles between each pair of genomes.

**Table S1.** Predicted polymorphic ratio of SINE insertions from different subfamilies located in intergenic and intragenic regions.

| Name of SINE subfamily (age) | Intragenic SINE insertions | | | |  | Intergenic SINE insertions | | |
| --- | --- | --- | --- | --- | --- | --- | --- | --- |
|  | No. of blast insertions | No. of predicted  polymorphic insertions | | The ratio of polymorphisms |  | No. of blast insertions | No. of predicted  polymorphic insertions | The ratio of polymorphisms |
| SINEA1 （2Ma） | 200 | 45 | 22.5 | |  | 200 | 53 | 26.5 |
| SINEA2 （5Ma） | 200 | 18 | 9.0 | |  | 200 | 21 | 10.5 |
| SINEA3 （5Ma） | 200 | 10 | 5.0 | |  | 200 | 25 | 12.5 |
| SINEA4 （11Ma） | 200 | 4 | 2.0 | |  | 200 | 1 | 0.5 |
| SINEB2 （23Ma） | 200 | 0 | 0.0 | |  | 200 | 0 | 0.0 |
| SINEB6 （27Ma） | 200 | 0 | 0.0 | |  | 200 | 1 | 0.5 |
| SINEC4 （32Ma） | 200 | 0 | 0.0 | |  | 200 | 0 | 0.0 |

**Table S2.** Polymorphic ratio of randomly selected polymorphic and non-polymorphic SINE insertions following PCR verification.

| SINE type | No. of randomly selected polymorphic or non-polymorphic insertions | | | | |  |
| --- | --- | --- | --- | --- | --- | --- |
|  | Predicted polymorphic by Blast | Confirmed polymorphic by PCR |  | Predicted non-polymorphic by Blast | Confirmed polymorphic by PCR | |
| SINEA1-A3 | 18 | 16 (83.33%) |  | 5 | 2 (40.00%) | |
| SINEA4-A11 | 4 | 4 (100.00%) |  | 9 | 2 (22.22%) | |
| SINEB | 1 | 1 (100.00%) |  | 2 | 0 (0.00%) | |
| SINEC | 2 | 1 (50.00%) |  | 9 | 0 (0.00%) | |
| Total | 25 | 22 (88.00%) |  | 25 | 4 (16.00%) | |

**Table S3.** Summary of the number of SINE insertions in the protocol used for annotating SINE RIPs.

| Genome | No. of SINE insertions | | | | |
| --- | --- | --- | --- | --- | --- |
|  | Repeatmasker result^1^ | Successfully mapped | Differential between genomes | Verified by Blast | Finally remained |
| Bama | 104260 | 100074 | 17641 | 6714 | 6603 |
| Bamei | 89114 | 86939 | 13555 | 4306 | 4306 |
| Berkshire | 92230 | 90415 | 9746 | 2856 | 2856 |
| Cross-bred | 102103 | 98412 | 9388 | 3054 | 2995 |
| Ellegaard Gottingen minipig | 81923 | 80073 | 13588 | 4222 | 4215 |
| Goettingen | 71314 | 68595 | 6395 | 736 | 736 |
| Hampshire | 93288 | 91538 | 9965 | 2994 | 2993 |
| Jinhua | 90567 | 88377 | 14522 | 4916 | 4915 |
| Landrace | 92016 | 90259 | 9991 | 2962 | 2961 |
| Large White | 91345 | 89580 | 10008 | 2893 | 2893 |
| Meishan | 88877 | 86708 | 14108 | 4714 | 4714 |
| Pietrain | 92023 | 90267 | 9984 | 2990 | 2990 |
| Rongchang | 88655 | 86424 | 13713 | 4426 | 4423 |
| Tibetan | 90277 | 87734 | 14916 | 4567 | 4536 |
| Wuzhishan | 91357 | 88602 | 15815 | 5272 | 5260 |
| Duroc | 103065 | 103065 | 80502 | 36452 | 13223 |
| Total | 1462414 | 1427062 | 263837 | 94074 | 70619 |

^1^: Repeatmasker result filtered with (1) SINEA1-3, (2) score≥1000, (3) length:100-330.

**Table S4.** Positive ratios of SINE RIPs obtained by PCR verification for rare SINE RIPs.

| Rare SINE RIPs type | No. of genome | No. of SINE RIPs be verified | No. of polymorphic sites | No. of Non- polymorphic sites | Positive ratio |
| --- | --- | --- | --- | --- | --- |
| Deletion | 2 | 28 | 9 | 19 | 32.14% |
|  | 3 | 32 | 12 | 20 | 37.50% |
|  | 4 | 32 | 26 | 6 | 81.25% |
| Insertion | 1 | 30 | 24 | 6 | 80.00% |
|  | 2 | 30 | 25 | 5 | 83.33% |
|  | 14 | 18 | 15 | 3 | 83.33% |
|  | 15 | 3 | 3 | 0 | 100.00% |

**Table S5.** Positive ratios of the 36,284 SINE RIPs obtained by PCR verification with limited samples.

| Source of SINE RIPs | Total No. of SINE RIPs | No. of SINE RIPs show polymorphic | Positive ratio | No. of uncertain SINE RIPs | No. of SINE RIPs show non-polymorphic |
| --- | --- | --- | --- | --- | --- |
| Meishan to Duroc | 27 | 24 | 88.89% | 2 | 1 |
| Duroc to Meishan | 28 | 22 | 78.57% | 4 | 2 |
| Landrace to Duroc | 30 | 24 | 80.00% | 4 | 2 |
| Duroc to Landrace | 28 | 23 | 82.14% | 3 | 2 |
| Large White to Duroc | 30 | 28 | 93.33% | 0 | 2 |
| Duroc to Large White | 30 | 18 | 60.00% | 9 | 3 |
| Duroc to Bama | 28 | 26 | 92.86% | 1 | 1 |
| Bama to Duroc | 29 | 20 | 68.97% | 7 | 2 |
| Total | 230 | 185 | 80.43% | 30 | 15 |

**Table S6.** Density of SINE RIPs in each chromosome.

| Chromosome | No. of SINE RIPs | Density(/1Mb) |
| --- | --- | --- |
| Chr1 | 3095 | 11.28** |
| Chr2 | 2139 | 14.08 |
| Chr3 | 1925 | 14.49 |
| Chr4 | 1902 | 14.53 |
| Chr5 | 1805 | 17.27 |
| Chr6 | 2247 | 13.15 |
| Chr7 | 2014 | 16.53 |
| Chr8 | 2076 | 14.94 |
| Chr9 | 2291 | 16.42 |
| Chr10 | 1500 | 21.63** |
| Chr11 | 1449 | 18.30* |
| Chr12 | 1195 | 19.40* |
| Chr13 | 2416 | 11.60** |
| Chr14 | 1972 | 13.91 |
| Chr15 | 1826 | 13.00 |
| Chr16 | 1169 | 14.62 |
| Chr17 | 1158 | 18.24 |
| Chr18 | 1047 | 18.70 |
| ChrX | 1610 | 12.78 |
| Others | 1448 | 13.14 |
| Total | 36284 | 14.50 |

Note:**/* means the density were different with total density with p<0.05/0.01 by Chi-square test.

**Table S7.** Characterization of 16 SINE RIPs analysed in 23 pig populations.

| Locus | Chr | Genotype frequency | | | Ne | Obs_Het | Exp_Het^a^ | PIC | F_IS_ | F_ST_ | No. of populations not comply with the Hardy–Weinberg equilibrium |
| --- | --- | --- | --- | --- | --- | --- | --- | --- | --- | --- | --- |
|  |  | +/+ | +/- | -/- |  |  |  |  |  |  |  |
| ESA1-98 | chr1 | 0.115 | 0.238 | 0.648 | 1.557 | 0.238 | 0.358 | 0.294 | 0.013 | 0.311 | 9 |
| REF-11172 | chr2 | 0.186 | 0.349 | 0.465 | 1.856 | 0.349 | 0.461 | 0.355 | 0.013 | 0.209 | 2 |
| REF-14902 | chr4 | 0.051 | 0.392 | 0.557 | 1.592 | 0.392 | 0.372 | 0.303 | -0.178 | 0.117 | 5 |
| DR-68328 | chr5 | 0.186 | 0.166 | 0.648 | 1.649 | 0.166 | 0.394 | 0.316 | 0.328 | 0.339 | 20 |
| REF-16266 | chr6 | 0.648 | 0.253 | 0.099 | 1.537 | 0.253 | 0.350 | 0.288 | -0.028 | 0.270 | 6 |
| REF-17668 | chr7 | 0.087 | 0.417 | 0.496 | 1.714 | 0.417 | 0.417 | 0.330 | -0.254 | 0.203 | 5 |
| ESA2-58 | chr8 | 0.487 | 0.395 | 0.118 | 1.760 | 0.395 | 0.432 | 0.339 | -0.195 | 0.221 | 4 |
| DR-93949 | chr9 | 0.113 | 0.327 | 0.561 | 1.666 | 0.327 | 0.400 | 0.320 | -0.177 | 0.284 | 4 |
| ESA1-16 | chr11 | 0.287 | 0.436 | 0.277 | 2.000 | 0.436 | 0.500 | 0.375 | -0.315 | 0.336 | 7 |
| REF-3992 | chr12 | 0.299 | 0.468 | 0.233 | 1.991 | 0.468 | 0.498 | 0.374 | -0.248 | 0.254 | 9 |
| ESA1-25 | chr13 | 0.530 | 0.332 | 0.139 | 1.734 | 0.332 | 0.423 | 0.334 | -0.049 | 0.241 | 6 |
| ESA2-18 | chr14 | 0.268 | 0.433 | 0.299 | 1.998 | 0.433 | 0.500 | 0.375 | -0.167 | 0.256 | 3 |
| ESA1-33 | chr15 | 0.149 | 0.268 | 0.583 | 1.683 | 0.268 | 0.406 | 0.323 | -0.037 | 0.369 | 3 |
| REF-9432 | chr16 | 0.192 | 0.448 | 0.361 | 1.944 | 0.448 | 0.486 | 0.368 | -0.057 | 0.128 | 6 |
| ESA1-42 | chr17 | 0.246 | 0.422 | 0.332 | 1.986 | 0.422 | 0.496 | 0.373 | -0.158 | 0.259 | 5 |
| ESA1-43 | chr18 | 0.079 | 0.323 | 0.598 | 1.575 | 0.323 | 0.365 | 0.298 | -0.183 | 0.242 | 6 |
| Mean |  | 0.245 | 0.354 | 0.401 | 1.765 | 0.354 | 0.429 | 0.335 | -0.106 | 0.252 | 6.250 |
| St. Dev |  | 0.173 | 0.088 | 0.193 | 0.172 | 0.088 | 0.055 | 0.031 | 0.153 | 0.069 | 4.155 |

a Nei's (1973) expected heterozygosity

| **Table S8. The pig genomes used for the SINE RIP screen protocol.** | | | | | | | | | | | |
| --- | --- | --- | --- | --- | --- | --- | --- | --- | --- | --- | --- |
| Genome Name | WGS Name | Size (Mb) | Scaffolds | Rele-ase Date | Level | Sequence depth | Sequence technology | Assembly method | Sex | Submitter | Website |
| Duroc （susScr11.1） | AEMK02 | 2501.91 | 706 | 2017-2-7 | Chromosome | 65.0x | PacBio | Falcon v. OCT-2015 | female | The Swine Genome Sequencing Consortium (SGSC) | https://www.ncbi.nlm.nih.gov/assembly/GCA_000003025.6 |
| Cross-bred(Yorkshire_Landrace_Duroc) | NPJO01 | 2755.44 | 14157 | 2017-12-20 | Chromosome | 65.0x | PacBio; Illumina NextSeq 500 | Celera Assembler v. 8.3rc2 | male | USDA ARS | https://www.ncbi.nlm.nih.gov/assembly/GCA_002844635.1 |
| Goettingen | LIDP01 | 2611.36 | 5206 | 2015-9-16 | Scaffold | 20.0x | 454; SOLiD | GMAP v. DEC-2012; BioScope v. 1.2; Newbler v. 2.7 | female | F. Hoffmann - La Roche AG | https://www.ncbi.nlm.nih.gov/assembly/GCA_001292865.1 |
| Wuzhishan | AJKK01 | 2508.91 | 137577 | 2015-3-18 | Scaffold | 120x | Illumina HiSeq2000 | SOAPdenovo v. 2.03 | male | BGI-shenzhen | https://www.ncbi.nlm.nih.gov/assembly/GCA_000325925.2 |
| Ellegaard Gottingen minipig | AOCR01 | 2358.02 | 231585 | 2013-1-10 | Contig | 79.0x | Illumina HiSeq | SOAPdenovo v. September 2011 | female | GlaxoSmithKline | https://www.ncbi.nlm.nih.gov/assembly/GCA_000331475.1 |
| Tibetan | AORO02 | 2437.74 | 72068 | 2016-8-8 | Scaffold | 131.0x | Illumina HiSeq | SOAPdenovo v. 2.0 | female | Novogene | https://www.ncbi.nlm.nih.gov/assembly/GCA_000472085.2 |
| Large White | LUXX01 | 2457.91 | 102342 | 2016-8-5 | Scaffold | 95.48x | Illumina HiSeq | SOAPdenovo v. 2.0 | female | Novogene | https://www.ncbi.nlm.nih.gov/assembly/GCA_001700135.1 |
| Rongchang | LUXR01 | 2459.03 | 120246 | 2016-8-5 | Scaffold | 93.54x | Illumina HiSeq | SOAPdenovo v. 2.0 | female | Novogene | https://www.ncbi.nlm.nih.gov/assembly/GCA_001700155.1 |
| Hampshire | LUXS01 | 2437.11 | 82206 | 2016-8-5 | Scaffold | 100.88x | Illumina HiSeq | SOAPdenovo v. 2.0 | female | Novogene | https://www.ncbi.nlm.nih.gov/assembly/GCA_001700165.1 |
| Meishan | LUXQ01 | 2467.5 | 133833 | 2016-8-5 | Scaffold | 111.13x | Illumina HiSeq | SOAPdenovo v. 2.0 | female | Novogene | https://www.ncbi.nlm.nih.gov/assembly/GCA_001700195.1 |
| Landrace | LUXT01 | 2440.98 | 94659 | 2016-8-5 | Scaffold | 91.23x | Illumina HiSeq | SOAPdenovo v. 2.0 | female | Novogene | https://www.ncbi.nlm.nih.gov/assembly/GCA_001700215.1 |
| Bamei | LUXV01 | 2460.76 | 129335 | 2016-8-5 | Scaffold | 88.72x | Illumina HiSeq | SOAPdenovo v. 2.0 | female | Novogene | https://www.ncbi.nlm.nih.gov/assembly/GCA_001700235.1 |
| Bama | SIDA01 | 2491.05 | 6610 | 2019-8-1 | Chromosome | 361x | Illumina; PacBio; Oxford Nanopore MinION; 10x Genomics; Hi-C | supernovo v. Dec-2016; SOAPdenovo v. Jan-2017; PBJelly v. Feb-2017; LACHESIS v. Feb-2017 | male | Novogene | https://www.ncbi.nlm.nih.gov/assembly/GCA_007644095.1 |
| Pietrain | LUXU01 | 2438.32 | 88436 | 2016-8-5 | Scaffold | 96.22x | Illumina HiSeq | SOAPdenovo v. 2.0 | female | Novogene | https://www.ncbi.nlm.nih.gov/assembly/GCA_001700255.1 |
| Jinhua | LUXY01 | 2453.7 | 115554 | 2016-8-5 | Scaffold | 109.05x | Illumina HiSeq | SOAPdenovo v. 2.0 | female | Novogene | https://www.ncbi.nlm.nih.gov/assembly/GCA_001700295.1 |
| Berkshire | LUXW01 | 2434.71 | 94468 | 2016-8-5 | Scaffold | 113.47x | Illumina HiSeq | SOAPdenovo v. 2.0 | female | Novogene | https://www.ncbi.nlm.nih.gov/assembly/GCA_001700575.1 |
